# A retrospective study of morbidity and mortality of chronic acid sphingomyelinase deficiency in Germany

**DOI:** 10.1186/s13023-024-03174-1

**Published:** 2024-04-13

**Authors:** Eugen Mengel, Nicole Muschol, Natalie Weinhold, Athanasia Ziagaki, Julia Neugebauer, Benno Antoni, Laura Langer, Maja Gasparic, Sophie Guillonneau, Marie Fournier, Fernando Laredo, Ruth Pulikottil-Jacob

**Affiliations:** 1 Institute of Clinical Science for LSD, SphinCS, Hochheim, Germany; 2 International Center for Lysosomal Disorders (ICLD), University Medical Center Hamburg-Eppendorf, Hamburg, Germany; 3 Department of Pediatric Gastroenterology, Nephrology and Metabolic Diseases, Center of Chronically Sick Children, Charité-Universitätsmedizin Berlin, Berlin, Germany; 4 Department of Endocrinology and Metabolism, Charité-Universitätsmedizin Berlin, Berlin, Germany; 5 IQVIA Commercial GmbH & Co. OHG, Frankfurt, Germany; 6 Sanofi, AHTC Building Amsterdam, Amsterdam, Netherlands; 7 Sanofi, Chilly-Mazarin, France; 8 Sanofi, São Paulo, Brazil; 9 Sanofi, Reading, UK

**Keywords:** Acid sphingomyelinase deficiency, Morbidity, Mortality, Overall survival, Standardised mortality ratio

## Abstract

**Background:**

Acid sphingomyelinase deficiency (ASMD) is a rare, progressive, potentially fatal lysosomal storage disease that exhibits a broad spectrum of clinical phenotypes. There is a need to expand the knowledge of disease mortality and morbidity in Germany because of limited information on survival analysis in patients with chronic ASMD (type B or type A/B).

**Methods:**

This observational, multicentre, retrospective cohort study was conducted using medical records of patients with the first symptom onset/diagnosis of ASMD type B or type A/B between 1st January 1990 and 31st July 2021 from four German medical centres. Eligible medical records were abstracted to collect data on demographic characteristics, medical history, hospitalisation, mortality, and causes of death from disease onset to the last follow-up/death. Survival outcomes were estimated using the Kaplan–Meier analysis. Standardised mortality ratio (SMR) was also explored.

**Results:**

This study included 33 chart records of patients with ASMD type B (*n* = 24) and type A/B (*n* = 9), with a median (interquartile range [IQR]) age of 8.0 [3.0–20.0] years and 1.0 [1.0–2.0] years, respectively, at diagnosis. The commonly reported manifestations were related to spleen (100.0%), liver (93.9%), and respiratory (77.4%) abnormalities. Nine deaths were reported at a median [IQR] age of 17.0 [5.0–25.0] years, with 66.7% of overall patients deceased at less than 18 years of age; the median [IQR] age at death for patients with ASMD type B (*n* = 4) and type A/B (*n* = 5) was 31.0 [11.0–55.0] and 9.0 [4.0–18.0] years, respectively. All deaths were ASMD-related and primarily caused by liver or respiratory failures or severe progressive neurodegeneration (two patients with ASMD type A/B). The median (95% confidence interval [CI]) overall survival age since birth was 45.4 (17.5–65.0) years. Additionally, an SMR [95% CI] analysis (21.6 [9.8–38.0]) showed that age-specific deaths in the ASMD population were 21.6 times more frequent than that in the general German population.

**Conclusions:**

This study highlights considerable morbidity and mortality associated with ASMD type B and type A/B in Germany. It further emphasises the importance of effective therapy for chronic ASMD to reduce disease complications.

**Supplementary Information:**

The online version contains supplementary material available at 10.1186/s13023-024-03174-1.

## Background

Acid sphingomyelinase deficiency (ASMD), historically known as Niemann–Pick disease types A, A/B, and B, is a rare lysosomal storage disease resulting from the deficiency of lysosomal enzyme acid sphingomyelinase (ASM) due to pathogenic variants in the *sphingomyelin phosphodiesterase 1* (*SMPD1*) gene [1, 2]. The deficiency of ASM, which catalyses the hydrolysis of sphingomyelin to ceramide and phosphocholine, leads to the progressive accumulation of sphingomyelin and other lipids within tissues, including the spleen, liver, lungs, bone marrow, and lymph nodes and may also affect neurons [3]. ASMD is pan-ethnic and affects males and females equally, with an estimated worldwide prevalence of 0.4–0.6 cases per 100,000 live births across gender or ethnicity [4]. However, the true incidence and prevalence of ASMD are likely underestimated because of a lack of disease awareness and underdiagnosis.

This disease presents with a wide range of clinical manifestations characterised by a phenotypic spectrum that is broadly divided into three subtypes: type A (infantile neurovisceral), type A/B (chronic neurovisceral) and type B (chronic visceral) [5]. Patients with ASMD type A experience onset in early infancy with rapidly progressive systemic manifestations and neurodegeneration, leading to death by the age of 3 years [2]. Patients presenting with slower neurological (e.g. gross motor delay, ataxia, and learning disability) and visceral disease progression and longer survival compared to ASMD type A are categorised as those with ASMD type A/B intermediate type [6–8]. Patients with ASMD type B show symptom onset from infancy to adulthood, with gradual progression of visceral manifestations without significant neurodegeneration [1]. Both ASMD type B and type A/B exhibit common multisystemic clinical manifestations, including splenomegaly, interstitial lung disease, hepatomegaly, liver dysfunction, dyslipidaemia, thrombocytopenia, heart disease, skeletal abnormalities and growth delays, shortness of breath, fatigue, and pain [9]. Overlapping symptoms with other chronic conditions often lead to delays in diagnosis, which is confirmed by demonstration of low ASM enzyme activity [10].

Survival outcomes among patients with chronic ASMD can vary widely [8]. Neurodegeneration (23.1%), respiratory failure (23.1%), and liver disease (19.2%) are the common causes of death in patients with ASMD type A/B, while respiratory (30.9%) and liver diseases (29.1%) are leading causes of death in type B [3, 8, 10]. In 2022, olipudase alfa (Xenpozyme®; Sanofi), a recombinant human ASM, was approved as the first enzyme replacement therapy for treating non-central nervous system manifestations of ASMD in paediatric and adult patients in more than 30 countries, including Japan, the European Union countries, and the United States [11, 12]. Olipudase alfa was reported to be well-tolerated during 2 years of treatment in children with ASMD type B or type A/B [13–15].

The substantial burden of ASMD presents an unmet need to better understand survival outcomes in patients with the disease. The current understanding of morbidity and mortality of ASMD is based on limited single-case reports, case series, and observational studies [16–18]. According to our knowledge, survival analyses of 103 patients with ASMD type B in the United States were first reported by McGovern et al., suggesting 17.5% deaths, with a median [range] age at death of 17.0 [2.0–72.0] years [19]. Thus, there is a need to analyse ASMD-related morbidity and mortality in the European Union.

This retrospective study aimed to evaluate the morbidity and mortality of patients with ASMD (across age and disease type A/B or type B) in Germany.

## Methods

### Study design

This observational, multicentre, retrospective cohort study was conducted using medical records from four German medical sites. The participating physicians abstracted data related to the survival history of patients with ASMD from childhood, adolescence, and adulthood. Patients with ASMD type B or type A/B with retrievable information from hospital records between 1st January 1990 and 31st July 2021 were included in this study. ASMD diagnosis was based on a low activity of ASM (< 10%). Patients with ASMD type A or those without retrievable information from the medical record were excluded.

The primary objective of the study was to estimate the survival probability in German patients with ASMD type B. The secondary objective was to describe the characteristics of patients with ASMD according to the subtype (type B or type A/B). This study also explored the survival of patients with ASMD type A/B.

This study was conducted in accordance with the Declaration of Helsinki and all subsequent amendments, the guidelines for Good Epidemiological Practice, and Good Pharmacoepidemiology Practices issued by the International Society for Pharmacoepidemiology. All study documents were reviewed by the leading Ethics Committee (Landesärztekammer Hessen) and the local Ethics Committee (Ärztekammer Hamburg). Sites located in Berlin did not require additional local review. All patients and/or patients’ parents/legal guardians provided informed consent or assent to participate in the study. For data collection from deceased patients, an informed consent waiver was used.

### Data collection

Data were retrospectively extracted from patient’s medical records during the observation period, defined as the period from the first evidence of ASMD to either the date of the last follow-up or end of the study period or death. The index date was the first date of symptom onset or diagnosis of the disease, whichever occurred first.

Data on demographic characteristics at the first symptom onset or diagnosis and at the last follow-up/death, medical history, laboratory examination (including liver function tests), and mortality (age and causes of death) were retrieved from eligible records. Clinical findings included any complications or events recorded in patient charts during the observation period. Data on healthcare resource use (due to ASMD-related complications), including hospitalisation, inpatient stays, outpatient visits, and emergency room visits, were analysed.

### Statistical analyses

Demographic characteristics and medical and development history at the date of first symptom onset, diagnosis, and last follow-up or death were characterised using descriptive statistics. Continuous variables were summarised using several non-missing and missing observations and represented as mean, standard deviation (SD), and median (interquartile range [IQR]). Categorical variables were summarised by frequency counts (*n*) and percentages (%). The unadjusted annualised clinical finding rate (in patient-years) was defined as:

$$\begin{aligned}& {\rm{The}}\,{\rm{unadjusted}}\,{\rm{annualised}}\,{\rm{rate}}\,  \\ & \quad =\frac{{\Sigma {\rm{number}}\,{\rm{of}}\,{\rm{events}}\,{\rm{during}}\,{\rm{the}}\,{\rm{observation}}\,{\rm{period}}}}{{\Sigma {\rm{number}}\,{\rm{of}}\,{\rm{patient}} - {\rm{years}}\,{\rm{on}}\,{\rm{study}}}}\end{aligned}$$

where Σnumber of patient-years is the sum (length of observation periods [years] of all patients).

Survival outcomes were estimated using Kaplan–Meier (KM) analysis. Overall survival (OS) was defined as the duration from birth until death due to any cause (event), considering the condition being present since birth, with age as the time scale. Data from patients who were alive were censored at the date of the last follow-up (end of the study observation period). Left truncation (i.e. individuals who died before being diagnosed with ASMD and were not accounted for in the study) might occur which may introduce a survival bias. To address this, adjusted survival analysis for the time from birth to the index date was conducted as a *post-hoc* analysis. The median OS with two-sided 95% confidence intervals (CIs) was evaluated.

Owing to the small sample size and a high proportion of censoring, standardised mortality ratio (SMR) was also assessed *post-hoc* to calculate survival probability. The expected number of deaths based on the age-specific mortality rates in the general population was retrieved from the German life Table [20] and applied to the number at risk in the ASMD database. The SMR for ASMD type B and type A/B population was calculated using the following formula:

$$ \text{S}\text{M}\text{R}=\frac{\text{A}\text{c}\text{t}\text{u}\text{a}\text{l}\, \text{n}\text{u}\text{m}\text{b}\text{e}\text{r}\, \text{o}\text{f}\, \text{d}\text{e}\text{a}\text{t}\text{h}\text{s}}{\text{E}\text{x}\text{p}\text{e}\text{c}\text{t}\text{e}\text{d}\, \text{n}\text{u}\text{m}\text{b}\text{e}\text{r}\, \text{o}\text{f}\, \text{d}\text{e}\text{a}\text{t}\text{h}\text{s}}$$

An SMR greater than one indicated an increased risk of mortality in the study population. As a measure of precision, 95% CI around the SMR estimate was also calculated. Survival probabilities for patients with ASMD were estimated using the calculated SMR in conjunction with the general population mortality data and represented graphically.

SMR computation was performed using Microsoft Excel (version 2208). All other analyses were conducted using SAS statistics software version 9.4 (SAS Institute Inc., Cary, NC, USA). All data were evaluated as reported, without any imputation for missing values.

## Results

### Patient characteristics

Overall, 33 patients were enrolled, of which 24 (72.7%) were classified as ASMD type B and 9 (27.3%) as ASMD type A/B (Table 1). All (100%) screened patients were enrolled as they met the inclusion criteria. The median [IQR] length of follow-up was 11.0 [5.0–20.0] years (Table 1). The index date was available for 32 (97.0%) out of the 33 patients.

**Table 1:** Patient disposition

Parameters	Statistics
Screened population [*n*]	33
Enrolled population [*n* (%)]^a^	33 (100.0)
**Length of follow-up (years)**	
Number	32
Mean (SD)	13.4 (11.3)
Median [IQR]	11.0 [5.0–20.0]
Range	0–44.0
Missing^b^	1
Completed the study [*n* (%)]	33 (100.0)

^a^Percentages were calculated using the number of screened patients as the denominator

^b^No information was reported

IQR, interquartile range; *n*, number of patients; SD, standard deviation

Patient demographics and clinical characteristics are presented in Table 2. The proportion of female patients with ASMD type B was 50.0% and that with ASMD type A/B was 66.7%. The median [IQR] age in the overall study population at the first evidence of ASMD and diagnosis was 2.0 [1.0–5.0] years (mean [SD]: 7.0 [11.8] years) and 4.0 [2.0–18.0] years (mean [SD]: 11.5 [14.3] years), respectively. The median [IQR] age at diagnosis for patients with ASMD type B was 8.0 [3.0–20.0] years and for those with ASMD type A/B was 1.0 [1.0–2.0] years. The median [IQR] time between first symptom onset and diagnosis for overall patients was 9.0 [1.0–89.0] months (Table 2).

**Table 2:** Patient baseline characteristics

Parameter	Overall (*N* = 33)	ASMD type B (*n* = 24)	ASMD type A/B (*n* = 9)
**Age at the first evidence of ASMD (years)**			
Number^a^	32	24	8
Mean (SD)	7.0 (11.8)	9.0 (13.1)	1.2 (1.2)
Median [IQR]	2.0 [1.0–5.0]	3.0 [2.0–15.0]	1.0 [1.0–1.0]
Range	0–58.0	0–58.0	0–4.0
**Age at diagnosis (years)**			
Number^a^	32	24	8
Mean (SD)	11.5 (14.3)	14.8 (15.2)	1.5 (1.1)
Median [IQR]	4.0 [2.0–18.0]	8.0 [3.0–20.0]	1.0 [1.0–2.0]
Range	1.0–58.0	1.0–58.0	1.0–4.0
**Time between the first symptom onset and diagnosis of ASMD (months)**			
Number^a^	29	22	7
Mean (SD)	58.7 (94.1)	76.1 (102.4)	4.3 (4.9)
Median [IQR]	9.0 [1.0–89.0]	21.0 [4.0–120.0]	1.0 [0–9.0]
Range	0–383	0–383	0–11
**Sex [** ***n*** **(%)]**			
Male	15 (45.5)	12 (50.0)	3 (33.3)
Female	18 (54.5)	12 (50.0)	6 (66.7)

^a^Patient with ASMD type A/B (*n* = 1) was excluded from the analysis due to significant missing variables

ASMD, acid sphingomyelinase deficiency; IQR, interquartile range; *n*, number of patients; SD, standard deviation

### Mortality analysis

At the time of data collection, 9 of 33 (27.3%) patients with ASMD (type B [*n* = 4, 16.7%] and type A/B [*n* = 5, 55.6%]) died (Table 3). The median [IQR] age at the time of death for patients with chronic ASMD was 17.0 [5.0–25.0] years; the median [IQR] age for patients with ASMD type A/B (9.0 [4.0–18.0] years) was lower than that for patients with ASMD type B (31.0 [11.0–55.0] years).

**Table 3:** Survival status of patients with ASMD type B or type A/B

Parameter	Overall (*N* = 33)	ASMD type B (*n* = 24)	ASMD type A/B (*n* = 9)
**Survival status at the time of data extraction [n (%)]** ^**a**^			
Survivors	24 (72.7%)	20 (83.3%)	4 (44.4%)
**Age at death (years)**			
Number	9	4	5
Mean (SD)	21.2 (21.1)	33.1 (27.1)	11.7 (9.2)
Median [IQR]	17.0 [5.0–25.0]	31.0 [11.0–55.0]	9.0 [4.0–18.0]
Range	3.0–65.0	5.0–65.0	3.0–25.0
**Cause of death (related to ASMD) [** ***n*** **(%)]** ^**b**^			
Liver disease^c^	2 (22.2)	1 (25.0)	1 (20.0)
Respiratory disease (respiratory failure/ insufficiency, pneumonia, pulmonary embolism, other, not known)	2 (22.2)	1 (25.0)	1 (20.0)
Severe progressive neurodegeneration	2 (22.2)	0	2 (40.0)
Bleeding	1 (11.1)	1 (25.0)	0
Multi-organ failure	1 (11.1)	1 (25.0)	0
Other^d^	1 (11.1)	0	1 (20.0)

^a^Percentages were calculated using the total number of patients with non-missing information on the analysed parameter as a denominator

^b^Percentages were calculated using the total number of deceased patients with non-missing information on the analysed parameter as a denominator

^c^The specific category of liver disease as the primary cause of death was not evaluated. However, the cause of liver disease was ASMD-related (liver failure by progressive liver fibrosis) and not acute toxic or inflammatory liver disease or malignant liver disease

^d^The cause of death was specified as acute respiratory distress syndrome, followed by hydropic decompensation of liver cirrhosis

ASMD, acid sphingomyelinase deficiency; IQR, interquartile range; *n*, number of patients; SD, standard deviation

**Fig. 1:**
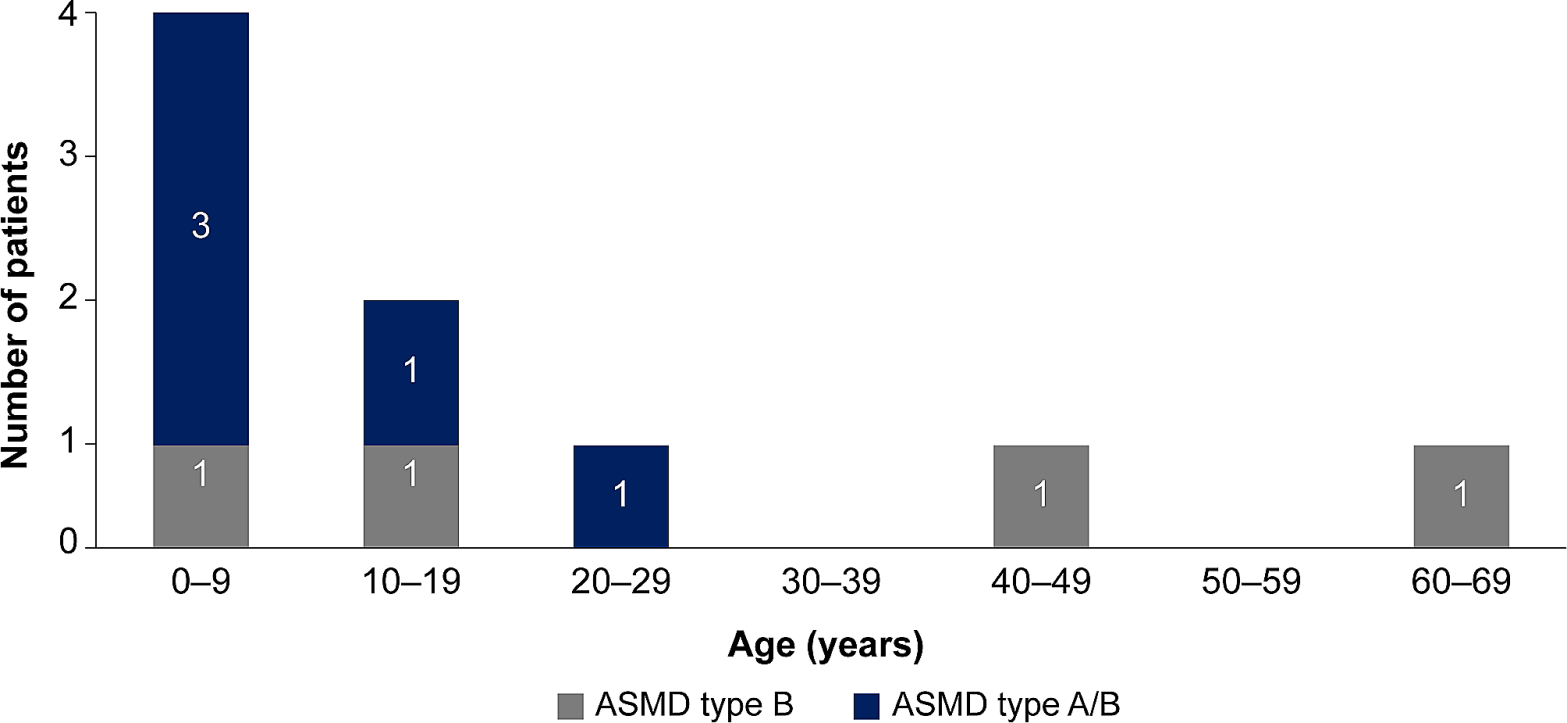
Distribution of patients with ASMD type B or type A/B based on age category at death. The numbers are based on patients with deceased status at the time of data extraction. ASMD, acid sphingomyelinase deficiency

A total of six (66.7%) deaths were reported in patients at < 18 years of age; two of these deaths occurred in patients with ASMD type B and four in those with ASMD type A/B (Fig. 1).

All deaths were reported to be related to ASMD (Table 3). The primary causes of death related to ASMD type B were liver disease (liver failure by progressive liver fibrosis), respiratory disease, bleeding, and multi-organ failure (25.0% each), whereas those related to ASMD type A/B were severe progressive neurodegeneration (40.0%), followed by liver disease (liver failure by progressive liver fibrosis), respiratory disease, and others (acute respiratory distress syndrome, followed by hydropic decompensation of liver cirrhosis) (20.0% each).

**Fig. 2:**
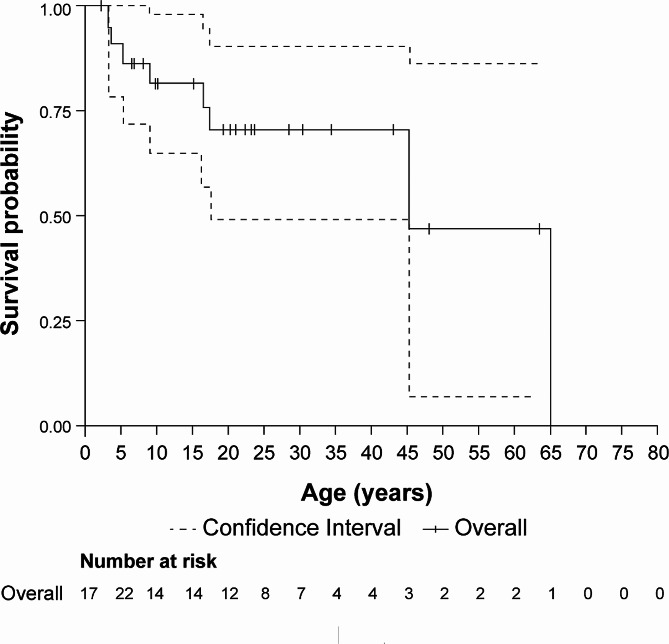
Kaplan–Meier survival curve (adjusted) from birth in patients with ASMD type B or type A/B. A deceased patient with ASMD type A/B could not be considered because the patient did not have the date for the first presentation of the disease. ASMD, acid sphingomyelinase deficiency; CI, confidence interval

The OS since birth was adjusted for left truncation based on the time from birth till the first evidence to account for the survival bias. The median (95% CI) OS time was 45.4 (17.5–65.0) years since birth for the overall ASMD cohort, with 75.0% of the data being censored (Fig. 2).

**Fig. 3:**
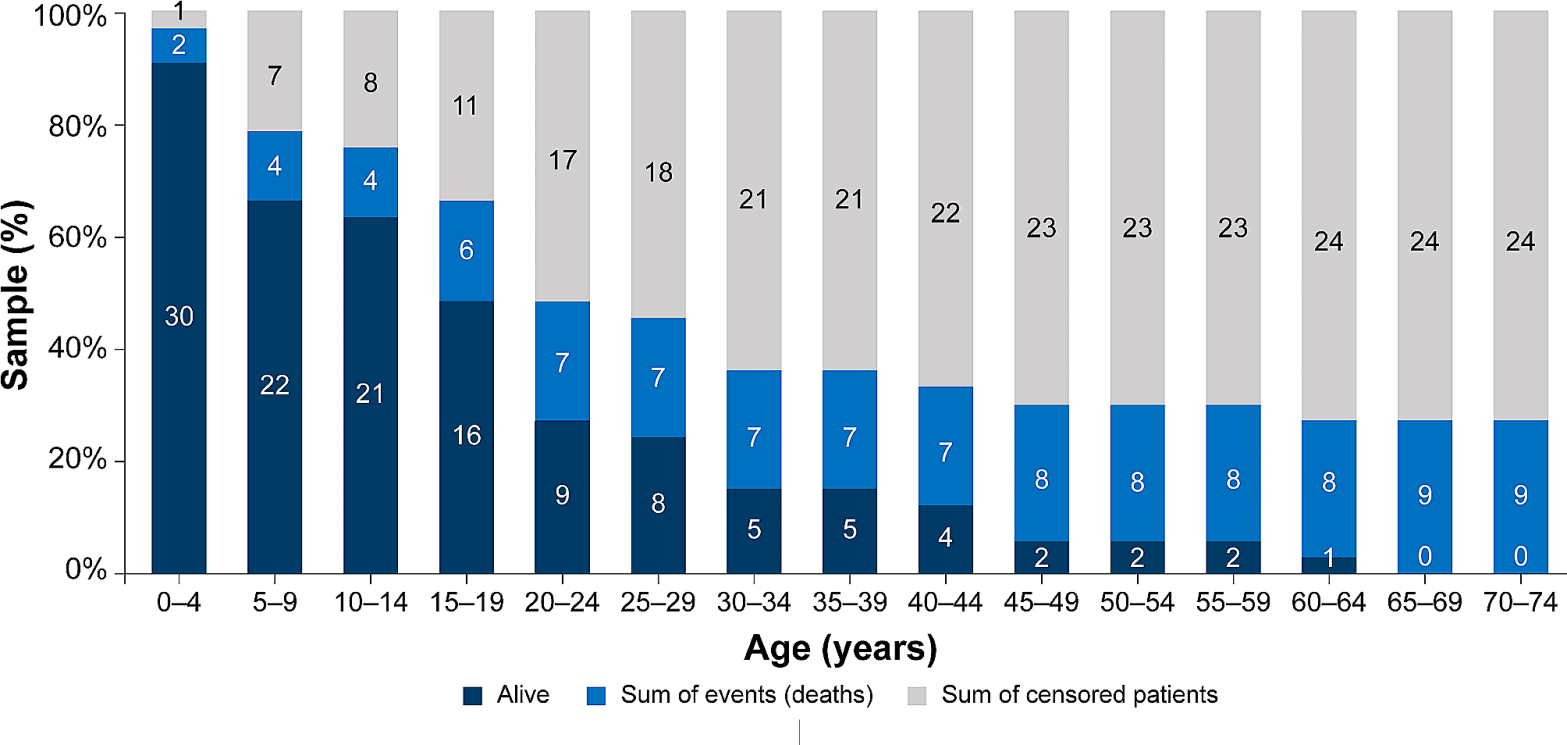
Sample breakdown of data used for survival analysis. The number of patients in a graph representing cumulative deaths and censored patients over time is based on the unadjusted KM curve. KM, Kaplan–Meier

As depicted in Fig. 3, a substantial proportion of the cohort (21/33, 63.6%) were censored by the age of 35, leaving only five patients at risk. The extensive censoring was likely because of young age of surviving patients after the end of the observation period. Therefore, in addition to the KM estimator, the SMR approach was used to address the potential limitations of estimating the mortality rate.

**Fig. 4:**
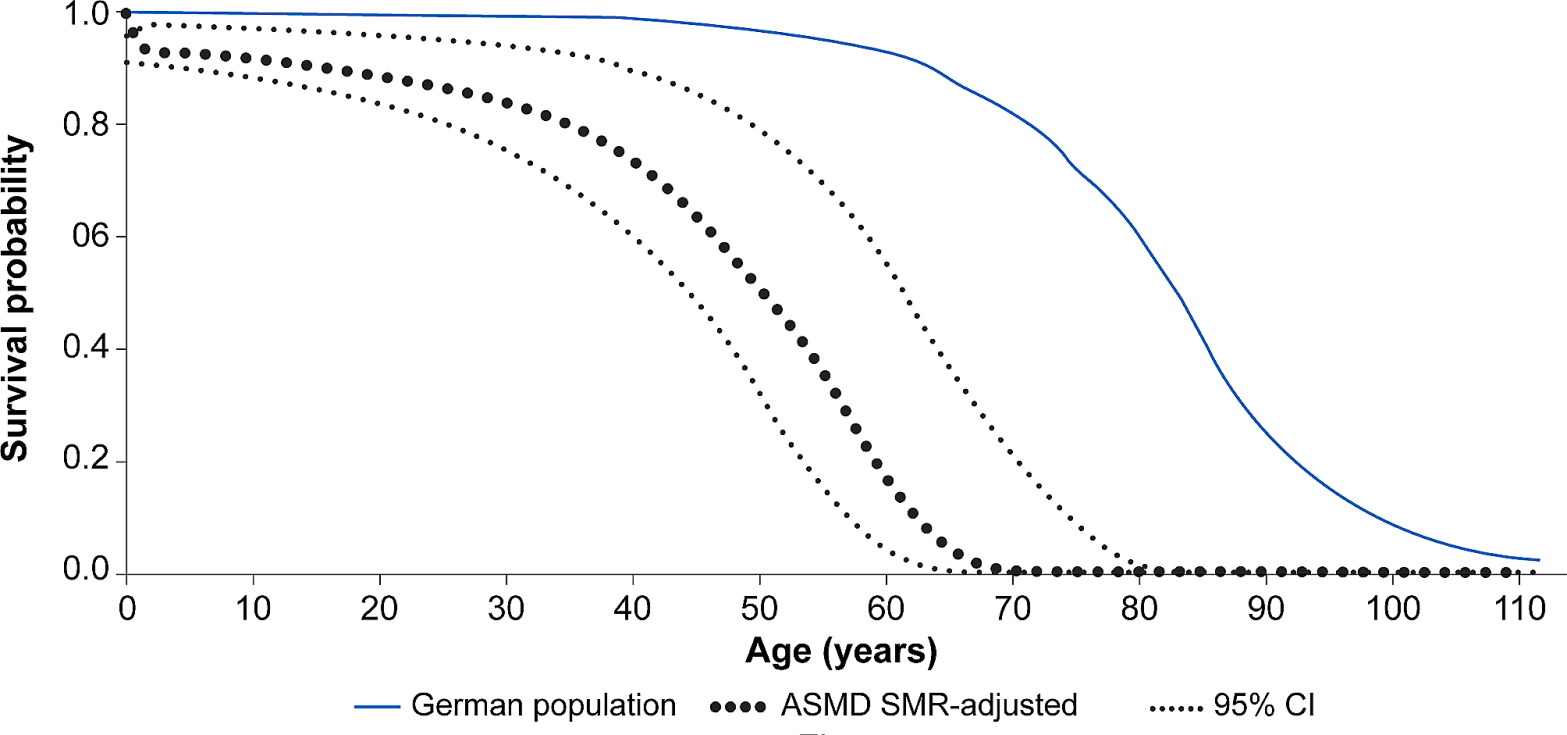
Survival probabilities for patients with ASMD type B and type A/B versus the general German population. The SMR calculation was based on the number of deceased patients (*n* = 9, including the patient without the date for the first presentation of the disease). ASMD, acid sphingomyelinase deficiency; CI, confidence interval; SMR, the standardised mortality ratio

An SMR [95% CI] of 21.6 [9.8–38.0] was estimated for the study population; the wide CI reflected the small sample size (*n* = 9). The estimated SMR of 21.6 was applied to the mortality rates of the general German population as a multiplier to calculate adjusted survival probabilities, as shown in Fig. 4.

### Clinical findings

At the first symptom onset or diagnosis, all patients (*n* = 33, 100.0%) had at least one clinical finding or complication reported in their medical records. Splenic (100.0%, *n/N* = 32/32), liver (93.9%, *n/N* = 31/33), and respiratory (77.4%, *n/N* = 24/31) manifestations were mostly reported in the overall population. These trends remained similar across patients with ASMD type B or type A/B (Table 4).

**Table 4:** Clinical findings related to ASMD based on ASMD type

Parameter	Overall (*N* = 33)	ASMD type B (*n* = 24)	ASMD type A/B (*n* = 9)
**Splenic clinical findings** ^a^			
At least one clinical finding/complication^b, c^	32 (100.0)	24 (100.0)	8 (100.0)
**Type of splenic clinical findings [** ***n*** **(%)]** ^d, e^			
Number^f^	32	24	8
Splenomegaly	31 (96.9)	23 (95.8)	8 (100.0)
Hypersplenism	8 (25.0)	6 (25.0)	2 (25.0)
Splenic infarction	2 (6.3)	2 (8.3)	0
**Liver clinical findings** ^g^			
At least one clinical finding/complication	31 (93.9)	22 (91.7)	9 (100.0)
No clinical findings/complications	2 (6.1)	2 (8.3)	0
**Type of liver clinical finding [** ***n*** **(%)]** ^d, e^			
Number^f^	31	22	9
Hepatomegaly	30 (96.8)	22 (100.0)	8 (88.9)
Liver cirrhosis	1 (3.2)	0	1 (11.1)
Portal hypertension	2 (6.5)	2 (9.1)	0
Ascites	2 (6.5)	2 (9.1)	0
Other	2 (6.5)	2 (9.1)	0
**Respiratory clinical findings** ^h^			
At least one clinical finding/complication^i^	24 (77.4)	18 (75.0)	6 (85.7)
No clinical findings/complications	7 (22.6)	6 (25.0)	1 (14.3)
**Type of respiratory clinical finding [** ***n*** **(%)]** ^d, e^			
Number^f^	24	18	6
Interstitial lung disease	18 (75.0)	12 (66.7)	6 (100.0)
Alveolar infiltrates	3 (12.5)	1 (5.6)	2 (33.3)
Respiratory distress	4 (16.7)	1 (5.6)	3 (50.0)
Lower respiratory tract infection	9 (37.5)	7 (38.9)	2 (33.3)
Other	3 (12.5)	2 (11.1)	1 (16.7)

^a^Percentages were calculated using the total number of patients (*n* = 32) with available data

^b^Patients with splenectomy prior to the index date (defined as the first date of evidence of ASMD, either first symptom onset or diagnosis) were excluded

^c^Patient with ASMD type A/B (*n* = 1) was excluded from the analysis owing to missing data

^d^Percentages were calculated using the total number of patients with non-missing information on the analysed parameter as a denominator

^e^Multiple responses were possible

^f^Patients with any clinical findings for respective organs (spleen/liver/lungs)

^g^Percentages were calculated using the total number of patients (*n* = 33) with available data

^h^Percentages were calculated using the total number of patients (*n* = 31) with available data

^i^ASMD type A/B (*n* = 2) were excluded from the analysis due to missing data

ASMD, acid sphingomyelinase deficiency; *n*, number of patients

In addition, a surgical history of splenectomy was reported for one patient (3.0%, *n*/*N* = 1/33) with ASMD type A/B. Furthermore, among the subset of patients with liver function tests at the first date of ASMD evidence, 92.3% ( 12/13) patients showed at least one abnormal parameter (Supplementary Table 1). All 12 (100.0%) patients with data available on alanine aminotransferase (ALT) presented with abnormal values. Of the 12 patients with data available on aspartate aminotransferase (AST), 11 (91.7%) had abnormal values reported.

In addition, a few patients with ASMD also had cardiovascular clinical findings (*n* = 8; ventricular hypertrophy, cardiac valvular disease, arterial hypertension, and others) and external bleeding episodes (*n* = 13; prolonged bleeding time, increased tendency to bruise and others) (Supplementary Table 2).

The median [IQR] age at the first clinical evidence of ASMD for the overall population was 2 [1.0–5.0] years (Table 2). Results of splenic and liver findings/complications were also similar across all age categories (Supplementary Table 3). Hepatomegaly were the most common reason for medical visits across all age groups.

### Healthcare resource use

During the observation period, 19 (57.6%) patients had at least one hospitalisation, including an inpatient stay (Supplementary Table 4); 14 (58.3%) patients with ASMD type B and 5 (55.6%) with ASMD type A/B had at least one hospitalisation. Among 19 hospitalisation cases, 13 (68.4%) patients underwent hospitalisation related to ASMD clinical findings/complications.

## Discussion

This study characterises morbidity and mortality in patients with ASMD type B or type A/B in a German cohort. While a previous study by McGovern et al. reported a detailed description of the major morbidities and causes of death in patients with chronic ASMD in the United States [19], the current study adds data from a German cohort, highlighting substantial morbidity and mortality associated with ASMD. Most importantly, this study reports the survival analysis of patients stratified by the type of ASMD.

In this study population, the proportion of female patients (54.5%) and the mean age at the first symptom onset and diagnosis (7.0 and 11.5 years, respectively) were slightly higher than findings from previous prospective, cross-sectional survey studies on patients with chronic ASMD [21]. McGovern et al. reported a population of 47.5% female patients with chronic ASMD, and the mean age at the first symptom onset and diagnosis was 5.0 and 9.8 years, respectively [21].

High mortality was observed in this German population, with a mortality rate of 27.3%. Overall, 66.7% of the deceased patients were aged less than 18 years at death; this included four patients with ASMD type A/B and two with ASMD type B. The mean age at death for overall nine deceased patients was 21.2 years. A previous report [19] has reported a mean age at death of 25.0 years for patients with chronic ASMD. The mean age at death for patients with ASMD type A/B (11.7 years) was lower than that for patients with ASMD type B (33.1 years). Similar observation was reported earlier by Cassiman et al. [8].

The results are also consistent with reported causes of death in patients with ASMD type B or type A/B [8, 22]. Cassiman et al. reported the leading causes of death as respiratory and liver failure, irrespective of age among patients with ASMD type B or type A/B [8]. Other reported causes of death were bleeding complications (which might be related in some cases to liver failure) and complications from bone marrow transplants, multi-organ failure, heart failure, and liver cancer [9]. Pneumonia was reported as the most common cause of death in patients with chronic ASMD by McGovern et al., 2021 [22]. Similar causes of death were noted in the overall ASMD population in the current study, primarily liver disease (22.2%), respiratory disease (22.2%, including respiratory failure, insufficiency, pneumonia, pulmonary embolism, and others), severe progressive neurodegeneration (22.2%), bleeding (11.1%), multi-organ failure (11.1%), and others (11.1%). Severe progressive neurodegeneration (40.0%), liver disease (20.0%), respiratory failure (20.0%), and other causes (20.0%), were reported as the causes of death in patients with ASMD type A/B (*n* = 5). These results are consistent with those presented by Cassiman et al., suggesting neurodegenerative disease (23.1%) along with liver disease (19.2%) and respiratory failure (23.1%) are the primary causes of death in patients with ASMD type A/B [8].

The current study further highlights the high incidence of morbidity associated with the splenic, hepatic, and respiratory findings in patients with ASMD, similar to previous reports [5, 8, 22]. All patients had at least one reported clinical finding/complication during the observation period, and 57.6% had an ASMD-related hospitalisation indicating high disease severity. These findings underline the need for regular clinical assessment in patients with ASMD. Common disease-related morbidities included splenomegaly (96.9%), hepatomegaly (96.8%), and interstitial lung disease (75.0%). A previous report involving patients with ASMD type B and A/B reported similar disease-related morbidities, including splenomegaly (96.6%), hepatomegaly (91.4%), liver dysfunction (82.6%), and pulmonary disease (75.0%) [8]. Another study involving chronic ASMD has reported splenomegaly (78.0%) and hepatomegaly (73.0%) as the most common clinical findings at the initial visit [10, 21]. A study involving the Dutch and Belgian patient populations with ASMD reported interstitial abnormalities in the lungs in 81.3% of patients with ASMD [23]. In the current study, liver and respiratory manifestations were mostly reported in ASMD type B. McGovern et al. [19] also reported liver disease and pulmonary disease as major morbidities in ASMD type B. Thus, patients with chronic ASMD display wide phenotypic heterogeneity, including a broad spectrum of disease manifestations and severity levels [1].

For life-threatening conditions present since birth, the KM analysis might be prone to left truncation as only patients alive until diagnosed with ASMD were included in the study. Therefore, the KM curve with adjusted analysis was performed to estimate the survival probability to avoid an overestimated survival. By conditioning the survival estimate on the age at diagnosis, the adjusted analysis was conducted to avoid the bias and hence, to present a more accurate estimate of survival. Given the rarity of ASMD, alternative methods of survival analysis may be helpful to address the limitations associated with small sample size and heavy censoring [22]. The overall SMR (95% CI) in patients with ASMD type B and type A/B indicated that the German ASMD population had 21.6 times more deaths than the age-specific mortality rates in the general population. Despite the steady increase in German life expectancy [24], our results indicate a large unmet need for better treatment options for patients with ASMD.

It is imperative for healthcare providers, policymakers, and the ASMD community to recognise the substantial impact of ASMD. Efforts aimed at improving diagnosis and expanding access are essential to ensure timely intervention to improve quality and extend life expectancy of patients living with ASMD.

The following limitations should be considered while interpreting the findings from this study. The study relied on retrospective data collected from available medical records. The findings are based on secondary data not collected for the purposes of this study. The study had a relatively small sample size. Only seven patients were above 6 years of age at the first evidence of the disease, and nine patients with ASMD type A/B were included, thereby impacting the generalisability of the findings. Further, the available data on clinical parameters were sparse because of missing information in the medical records; this was attributed to a lack of unified medical record systems and patients seeking care at multiple facilities. Liver function tests were analysed only at the first date of evidence (follow-up data were not retrieved); they were not standardised, nor were they performed by a central laboratory, thereby introducing variability. Absence of clinical guidelines for monitoring or managing patients with ASMD type B and A/B during the study duration may have contributed to infrequent examinations and assessments; hence, some examinations and assessments might not have been regularly performed and reported, as currently recommended in recent guidelines [7, 25]. Additionally, hospitalisation records in local hospitals might not be well documented, leading to underreporting hospitalizations. The OS for patients with ASMD type B was based on a limited number of events (*n* = 4) during the observation time. Heavy censoring was noted in these patients by the age of 35 years, leaving only a few patients at risk. This study used SMR analysis as an alternative approach to estimate OS. However, its calculation also relies on the available information from the patient charts and assumes a constant increase in mortality risk across ages and might not accurately represent the shape of the survival function for patients with ASMD type B or type A/B.

## Conclusions

To our knowledge, this study is the first to examine the survival outcomes of patients with ASMD in Germany. This study provides compelling evidence that ASMD is a life-shortening disease, and ASMD type B or type A/B is associated with high morbidity and mortality, especially in the paediatric German population. The study also reports the primary causes of chronic ASMD-related death, which were predominantly linked to liver failure, respiratory failure, and neurodegeneration (only in ASMD type A/B). Presently, with the availability of an effective disease-modifying therapy, early diagnosis and treatment is of utmost importance in order to address and prevent disease complications.

### Electronic supplementary material

Below is the link to the electronic supplementary material.

Supplementary Material 1

## Data Availability

All data generated or analysed during this study are included in this article and its supplementary information files. Patient level data will be anonymised and study documents will be redacted to protect the privacy of the participants.

## Abbreviations

ASM: Acid sphingomyelinase
ASMD: Acid sphingomyelinase deficiency
CI: Confidence interval
ERT: Enzyme replacement therapy
KM: Kaplan–Meier
OS: Overall survival
SD: Standard deviation
SMR: Standardised mortality ratio
