# Supplementary material for: A retrospective study of morbidity and mortality of chronic acid sphingomyelinase deficiency in Germany
Source: Orphanet J Rare Dis. 2024 Apr 13;19:161. doi: 10.1186/s13023-024-03174-1 (PMC11015682; doi:10.1186/s13023-024-03174-1)
Supplement: Supplementary file 1 — Supplementary Material 1 [file 13023_2024_3174_MOESM1_ESM.docx]

**Supplementary Table 1** Liver function laboratory tests at the first date of evidence of ASMD^a^

| **Parameter** | **Overall  (*N* = 33)** | **ASMD type B  (*n* = 24)** | **ASMD type A/B (*n* = 9)** |
| --- | --- | --- | --- |
| **Liver function laboratory test** | | | |
| Number^b^ | 13 | 7 | 6 |
| At least one abnormal parameter [*n* (%)] | 12 (92.3) | 6 (85.7) | 6 (100.0) |
| **ALT** | | | |
| Number | 12 | 6 | 6 |
| Abnormal ALT level [*n* (%)] | 12 (100.0) | 6 (100) | 6 (100.0) |
| **AST** | | | |
| Number | 12 | 6 | 6 |
| Abnormal AST level [*n* (%)] | 11 (91.7) | 5 (83.3) | 6 (100.0) |

^a^The closest laboratory test/examination within 12 months around the first date of evidence of ASMD was retained

^b^Patients with available information on liver function test data were included

Abnormal parameter was according to the reference values of the local laboratories as reported by the participating investigators

ASMD, acid sphingomyelinase deficiency; ALT, alanine aminotransferase; AST, aspartate aminotransferase; *n*, number of patients

**Supplementary Table 2** Other clinical findings related to ASMD type

| **Parameter** | **Overall**  **(*N* = 33)** | **ASMD type B**  **(*n* = 24)** | **ASMD type A/B**  **(*n* = 9)** |
| --- | --- | --- | --- |
| **Cardiovascular clinical finding** | | | |
| Number^a,b^ | 30 | 23 | 7 |
| At least one clinical finding/complication^a,b^ | 8 (26.7) | 7 (30.4) | 1 (14.3) |
| No clinical findings/complications | 22 (73.3) | 16 (69.6) | 6 (85.7) |
| **Type of cardiovascular clinical finding [*n* (%)]**^c,d^ | | | |
| Number^a^ | 8 | 7 | 1 |
| Ventricular hypertrophy | 1 (12.5) | 0 | 1 (100) |
| Cardiac valvular disease | 2 (25.0) | 2 (28.6) | 0 |
| Arterial hypertension | 4 (50.0) | 4 (57.1) | 0 |
| Other | 1 (12.5) | 1 (14.3) | 0 |
| **External bleeding episodes** | | | |
| Number^a,b^ | 30 | 23 | 7 |
| At least one clinical finding/complication^a,b^ | 13 (43.3) | 10 (43.5) | 3 (42.9) |
| No clinical findings/complications | 17 (56.7) | 13 (56.5) | 4 (57.1) |
| **Type of external bleeding episode [*n* (%)]**^c,d^ | | | |
| Number^a^ | 13 | 10 | 3 |
| Prolonged bleeding time | 2 (15.4) | 0 | 2 (66.7) |
| Increased tendency to bruise | 5 (38.5) | 4 (40.0) | 1 (33.3) |
| Other | 11 (84.6) | 9 (90.0) | 2 (66.7) |

^a^Patients with any clinical findings for respective types

^b^Patients were excluded from the analysis due to missing data

^c^Percentages were calculated using the total number of patients with non-missing information on the analysed parameter as a denominator

^d^Multiple responses were possible

ASMD, acid sphingomyelinase deficiency; *n*, number of patients

**Supplementary Table 3** Clinical findings related to ASMD based on age category

| **Parameter** | **Age category at the first clinical evidence (years)** | | |
| --- | --- | --- | --- |
|  | **0–5** | **6–17** | **≥18** |
| **Splenic clinical findings** | | | |
| At least one clinical finding/complication^a^ | 25 (100.0) | 2 (100.0) | 5 (100.0) |
| **Type of splenic clinical findings [*n* (%)]**^b,c^ | | | |
| Number | 25 | 2 | 5 |
| Splenomegaly | 24 (96.9) | 2 (100.0) | 5 (100.0) |
| Hypersplenism | 5 (20.0) | 0 | 3 (60.0) |
| Splenic infarction | 2 (8.0) | 0 | 0 |
| **Liver clinical findings** | | | |
| At least one clinical finding/complication | 25 (100.0) | 2 (100.0) | 5 (100.0) |
| **Type of liver clinical finding [*n* (%)]**^b,c^ | | | |
| Number | 24 | 1 | 5 |
| Hepatomegaly | 24 (100.0) | 1 (100.0) | 5 (100.0) |
| Portal hypertension | 2 (8.3) | 0 | 0 |
| Ascites | 1 (4.2) | 0 | 1 (20.0) |
| Other | 2 (8.3) | 0 | 0 |
| **Respiratory clinical findings** | | | |
| Number | 24 | 2 | 5 |
| At least one clinical finding/complication | 20 (83.3) | 2 (100.0) | 2 (40.0) |
| No clinical findings/complications | 4 (16.7) | 0 | 3 (60.0) |
| **Type of respiratory clinical finding [*n* (%)]**^b,c^ | | | |
| Number | 20 | 2 | 2 |
| Clinical features of interstitial lung disease | 17 (85.0) | 0 | 1 (50.0) |
| Alveolar infiltrates | 3 (15.0) | 0 | 0 |
| Respiratory distress | 4 (20.0) | 0 | 0 |
| Lower respiratory tract infection | 8 (40.0) | 1 (50.0) | 0 |
| Other | 1 (5.0) | 1 (50.0) | 1 (50.0) |

^a^Patients with splenectomy prior to the index date (defined as the first date of evidence of ASMD, either first symptom onset or diagnosis) were excluded

^b^Percentages were calculated using the total number of patients with non-missing information on the analysed parameter as a denominator

^c^Multiple responses were possible

ASMD, acid sphingomyelinase deficiency; *n*, number of patients

**Supplementary Table 4** Hospitalisation in patients with ASMD type B or type A/B during the observation period

| **Parameter** | **Overall  (*N* = 33)** | **ASMD type B  (*n* = 24)** | **ASMD type A/B (*n* = 9)** |
| --- | --- | --- | --- |
| **Any hospitalisation during the observation period [*n* (%)]** | | | |
| At least one hospitalisation | 19 (57.6) | 14 (58.3) | 5 (55.6) |
| No hospitalisation | 13 (39.4) | 10 (41.7) | 3 (33.3) |
| Unknown | 1 (3.0) | 0 | 1 (11.1) |
| **Type of hospitalisation [*n* (%)]** | | | |
| Number^a^ | 19 | 14 | 5 |
| Inpatient stay | 19 (100.0) | 14 (100.0) | 5 (100.0) |
| Emergency room visit | 3 (15.8) | 2 (14.3) | 1 (20.0) |
| ASMD-related clinical findings | 13 (68.4) | 8 (57.1) | 5 (100.0) |
| Any other | 12 (63.2) | 12 (85.7) | 0 |

^a^Patients with at least one hospitalisation during the observation period

ASMD, acid sphingomyelinase deficiency; *n*, number of patients; SD, standard deviation
